# Interpretable Trials: Is Interpretability a Reason Why Clinical Trials Fail?

**DOI:** 10.3389/fmed.2021.541405

**Published:** 2021-08-09

**Authors:** Yi-Sheng Chao, Chao-Jung Wu, Hsing-Chien Wu, Danielle McGolrick, Wei-Chih Chen

**Affiliations:** ^1^Independent Researcher, Montreal, QC, Canada; ^2^Département d'informatique, Université du Québec à Montréal, Montreal, QC, Canada; ^3^Taipei Hospital, Ministry of Health and Welfare, New Taipei City, Taiwan; ^4^Department of Chest Medicine, Taipei Veterans General Hospital, Taipei, Taiwan; ^5^Faculty of Medicine, School of Medicine, National Yang-Ming University, Taipei, Taiwan; ^6^Institute of Emergency and Critical Care Medicine, School of Medicine, National Yang Ming Chiao Tung University, Taipei, Taiwan

**Keywords:** clinical trials, indices, composite measure, interpretability, clinicaltrials.gov

## Abstract

**Background:** There are clinical trials using composite measures, indices, or scales as proxy for independent variables or outcomes. Interpretability of derived measures may not be satisfying. Adopting indices of poor interpretability in clinical trials may lead to trial failure. This study aims to understand the impact of using indices of different interpretability in clinical trials.

**Methods:** The interpretability of indices was categorized as: fair-to-poor, good, and unknown. In the literature, frailty indices were considered fair to poor interpretability. Body mass index (BMI) was highly interpretable. The other indices were of unknown interpretability. The trials were searched at clinicaltrials.gov on October 2, 2018. The use of indices as conditions/diseases or other terms was searched. The trials were grouped as completed, terminated, active, and other status. We tabulated the frequencies of frailty, BMI, and other indices.

**Results:** There were 263,928 clinical trials found and 155,606 were completed or terminated. Among 2,115 trials adopting indices or composite measures as condition or disease, 244 adopted frailty and 487 used BMI without frailty indices. Significantly higher proportions of trials of unknown status used indices as conditions/diseases or other terms, compared to completed and terminated trials. The proportions of active trials using frailty indices were significantly higher than those of completed or terminated trials.

**Discussion:** Clinical trial databases can be used to understand why trials may fail. Based on the findings, we suspect that using indices of poor interpretability may be associated with trial failure. Interpretability has not been conceived as an essential criterion for outcomes or proxy measures in trials. We will continue verifying the findings in other databases or data sources and apply this research method to improve clinical trial design. To prevent patients from experiencing trials likely to fail, we suggest further examining the interpretability of the indices in trials.

## Introduction

There are a variety of diseases or conditions assessed in clinical trials. Many of them are direct measurement of physical conditions or pathological diagnoses, i.e., survival status and blood pressure. In contrast, several of them could not be directly measured or quantified based on single variables. For example, frailty is theorized as a geriatric syndrome that can be defined by a composition of variables, from four to 70 depending on the theories used to support the definitions (1–6). Frailty is often calculated on a continuous scale and dichotomized to derive frailty status (1–3, 5, 7–11). It has also been associated with a variety of outcomes, such as falls and mortality (1–5, 8, 10, 11). In addition to serving as proxy measures for health status, frailty itself has been used as an outcome of interventions (1–3, 8–12). One of the reasons is that frailty status has been linked to pathological changes, such as sarcopenia (13). Frailty status has been conceived as an opportunity to shift the aging trajectory and is actively used in various trials (1).

However, there are issues related to the use of composite measures or indices, such as frailty indices and three diagnoses of mental illnesses in clinical trials (1, 14). One of the critical issues is interpretability. Three of the most widely used frailty indices have been found difficult to interpret for several reasons (1). First, they may be better explained by noise or bias introduced due to inadequate data processing (1). Body mass index (BMI) and three of the widely used frailty indices have been approximated with input variables, as well as the biases (1). While 99.4% of the BMI variances can be explained by its input variables, the bias can explain 71.9% of the variance of one frailty index (1). For the same index, the bias variables also better predict mortality than frailty status (1). Second, the frailty statuses are in fact the sum of continuous frailty indices and biases induced by variable dichotomization (1). It has been well-recognized that variable categorization can be associated with information bias (1, 15) and misclassification bias (16). Third, the threshold for dichotomization is also problematic. There are conflicting views about the choices of the cut-off thresholds for dichotomization. One theory suggests not to adopt common symptoms and the other requires at least 20% of the populations to be eligible for the frailty criteria (1, 8). The choice of thresholds for dichotomization is also related to the direction of bias (1). Lastly, the frailty index consisting of 70 variables in the initial theory can be further simplified with fewer numbers of input variables (1). This is because many input variables are likely to be correlated with each other and the sum of many correlated variables may not be more informative than a few of them (1).

The use of conditions or diseases that may not be fully interpretable in clinical trials warrants caution as the consequences are severe. If these composite conditions are used as proxy measures to predict the outcomes, researchers may be misled and ignore other factors that can better explain the outcomes, especially the input variables of the composite measures or indices (1, 7). It has been noticed that the input variables of the three frailty indices better predicted mortality than the indices (1). In addition, it is very likely that there are numerous alternative indices that can be mined and better predict outcomes (1, 17). If the conditions defined by indices are used as outcomes, the danger is that unnecessary interventions may be designed and tested among patients that should be treated otherwise (16). Taking metabolic syndrome as example, it requires the information on five conditions to confirm the diagnosis (18–20). However, it is later found not predictive of other important outcomes, such as cardiovascular disease and diabetes (21).

To understand the extent of the interpretability problem in clinical trials, we think it important to estimate the numbers of trials that may be involved in conditions that are not fully interpretable, as well as the impact of interpretability on the execution of clinical trials. This hypothesis-generating study aims to provide initial evidence regarding whether interpretability of terms in trials may be associated with early termination or suspension of clinical trials.

## Methods

The *clinicaltrials.gov* site was searched with the indices of different interpretability listed in Table 1. This site maintained information on clinical studies (22). This site was established due to the legislation in 1997 and was made public in 2000 (22). The scope of this site was expanded in 2007 and 2017 to include more types of trials (22). In addition to summary information of trials, the following sections of the trial protocols were included: diseases or conditions, interventions, titles, description, study design, requirements for participation (eligibility criteria), locations where the study was being conducted, contact information for the study locations, links to relevant information on other health Web sites (22). Sometimes the trial results were available, such as participant description, study outcomes, and adverse events (22).

**Table 1:** The distribution of indices of different interpretability across clinical trials of three statuses.

**Interpretability classification**	**Representative terms**	**Search strategies**	**Total (100%)**				**Active trials (28.2% of all trials)**				**Competed trials (53.3% of all trials)**				**Terminated trials (5.6% of all trials)**				**Trials of other statuses (12.7%)**			
			**Conditions or diseases**		**Other terms only**		**Conditions or diseases only**		**Other terms only**		**Conditions or diseases only**		**Other terms only**		**Conditions or diseases only**		**Other terms only**		**Conditions or diseases only**		**Other terms only**	
		Index OR indices OR composite measures OR frailty OR frailness OR body mass index	3000	(1.05%)	43210	(15.18%)	897	(1.12%)	15163	(18.89%)	1575	(1.04%)	20938	(13.81%)	101	(0.63%)	2463	(15.34%)	427	(1.18%)	4646	(12.85%)
		Frailty OR frailness	291	(0.10%)	263	(0.09%)	145	(0.18%)	144	(0.18%)	105	(0.07%)	189	(0.12%)	6	(0.04%)	13	(0.08%)	35	(0.10%)	22	(0.06%)
		(Frailty OR frailness) OR (body mass index)	545	(0.19%)	4016	(1.41%)	201	(0.25%)	1391	(1.73%)	258	(0.17%)	2085	(1.38%)	13	(0.08%)	129	(0.80%)	73	(0.20%)	411	(1.14%)
		Body mass index	254	(0.09%)	3773	(1.33%)	56	(0.07%)	1262	(1.57%)	153	(0.10%)	2158	(1.42%)	7	(0.04%)	116	(0.72%)	38	(0.11%)	390	(1.08%)
		(Frailty OR frailness) AND (body mass index)	0	(0.00%)	20	(0.01%)	0	(0.00%)	15	(0.02%)	0	(0.00%)	4	(0.00%)	0	(0.00%)	0	(0.00%)	0	(0.00%)	1	(0.00%)
Good	BMI only	Strategy (4–5)	254	(0.09%)	3753	(1.32%)	56	(0.07%)	1247	(1.55%)	153	(0.10%)	2154	(1.42%)	7	(0.04%)	116	(0.72%)	38	(0.11%)	389	(1.08%)
Fair to poor	Frailty	Strategy (2)	291	(0.10%)	263	(0.09%)	145	(0.18%)	144	(0.18%)	105	(0.07%)	189	(0.12%)	6	(0.04%)	13	(0.08%)	35	(0.10%)	22	(0.06%)
Unclear	Index, excluding BMI, and frailty	Strategy (1–3)	2455	(0.86%)	39194	(13.77%)	696	(0.87%)	13772	(17.15%)	1317	(0.87%)	18853	(12.44%)	88	(0.55%)	2334	(14.54%)	354	(0.98%)	4235	(11.71%)
Not applicable to the use of indices	Unrelated to index	Total–strategy (1)	281644	(98.95%)	241434	(84.82%)	79393	(98.88%)	65127	(81.11%)	150030	(98.96%)	130409	(86.02%)	15951	(99.37%)	13589	(84.66%)	35726	(98.82%)	31507	(87.15%)
Total			284644	(100.00%)	284644	(100.00%)	80290	(100.00%)	80290	(100.00%)	151605	(100.00%)	151605	(100.00%)	16052	(100.00%)	16052	(100.00%)	36153	(100.00%)	36153	(100.00%)

*p < 0.001 for the distribution of indices of different interpretability across four types of trials regarding the search in both conditions/diseases and other terms according to Chi-square tests (Chi-squared = 105.9 and 1283.6, respectively). Active trials are those “not yet recruiting,” “recruiting,” “enrolling by invitation,” and “active, not recruiting.” Other statuses are “suspended,” “withdrawn,” and “unknown.” Search date on clinicaltrials.gov: October 2, 2018*.

To demonstrate the interpretability issue, the numbers of the trials that included the above-mentioned indices as conditions/diseases or other terms were tabulated. Based on the evidence, (1) frailty indices were considered not adequately interpretable for matching the following criteria: more than 25% of variances explained by biases or measurement errors and (2) excessive numbers of redundant variables (1). Based on the criteria, frailty indices were classified as fair to poor interpretability (1). BMI was found to be highly interpretable (1). Other indices were considered to have unknown interpretability.

Two search strategies were allowed in this site: conditions/diseases or other terms. The conditions or diseases were defined as “the disease, disorder, syndrome, illness, or injury that is being studied” (23). Other terms were defined as a search feature that helped to narrow the search by looking for a name of a drug or the registration number of a clinical study (23).

Trials were classified into four categories based on recruitment status: completed, terminated, active, and other. The completed trials were the studies that had ended normally, and participants were “no longer being examined or treated (that is, the last participant's last visit has occurred)” (23). Terminated trials were those that had stopped early and would not start again (23). In terminated trials, participants were “no longer being examined or treated” (23). Active trials were those “not yet recruiting,” “recruiting,” “enrolling by invitation,” and “active, not recruiting” (23). Other statuses were “suspended,” “withdrawn,” and “unknown” (23).

### Estimation of the Interpretability of Clinical Trials by Types of Indices Used

**Figure 1:**
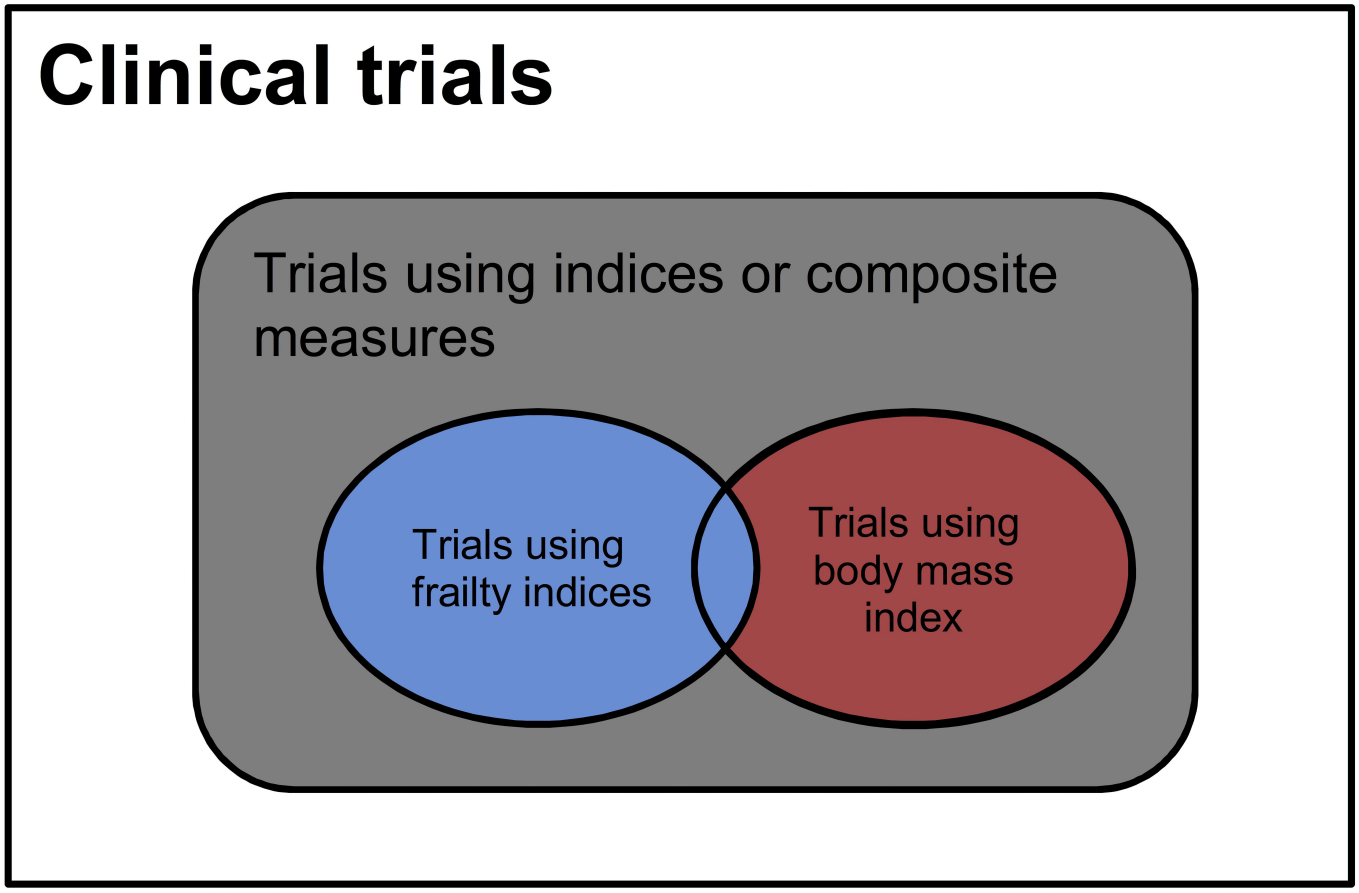
Distribution of clinical trials of different interpretability. Blue = trials involving the diseases or measures of poor to fair interpretability, red = trials using body mass index that is highly interpretable, black = trials involving the diseases or measures of unknown interpretability.

The numbers of clinical trials related to different types of indices were calculated based on the search terms in Table 1. The relationships between different search strategies were shown in Figure 1. The numbers of trials that involved any types of indices or composite measures were searched with indices or composite measures or frailty or BMI. The total numbers of the trials using BMI only, frailty indices, any indices other than frailty, or BMI were calculated based on these searches. The trials that used BMI only were considered using a condition that was highly interpretable (1). Those using frailty indices with or without BMI were of fair to poor interpretability (24). Those using indices other than frailty or BMI were of unknown interpretability. The percentages of the trials in relation to all trials were calculated. The distribution of trials of different interpretability was compared between types of search fields: conditions/diseases or other terms through chi-square tests. The associations between interpretability and trial status (completed, terminated, and other) were investigated with multinomial logit regression (25). Compared to using good-interpretability indices as diseases/conditions, the independent variables were (1) using fair-to-poor-interpretability indices as diseases/conditions, (2) using unclear-interpretability indices as diseases/conditions, (3) using good-interpretability indices for other terms, (4) using fair-to-poor-interpretability indices for other terms, (5) using unclear-interpretability indices for other terms, and (6) not using any indices or composite measures. The outcomes were trial statues: completed, terminated, and other statuses. The effect sizes were estimated with odds ratios (ORs). *P*-values < 0.05, two-tailed, were considered statistically significant. The statistical analysis was conducted with R (26) and RStudio (27).

## Results

There were 284,644 clinical trials identified on October 2, 2018. There were 80,290 active trials (28.2%), 151,605 completed trials (53.3%), 16,052 terminated trials (5.6%), and 36,153 trials of other status (12.7%). Among 3,000 trials that adopted any indices as conditions or diseases, 254 used BMI without frailty indices and were rated as trials involving interpretable conditions (0.09% of all trials). There were 291 trials using frailty indices as conditions that were rated as fair to poor interpretability (0.1%) and 2,455 using other types of indices of unknown interpretability (0.9%). The other 281,644 trials did not use any types of indices as conditions or diseases (98.95%).

There were respectively, 3,753 and 263 trials used BMI without frailty indices and frailty indices for terms apart from conditions or diseases (1.32 and 0.1% of all trials, respectively). There were 39,194 trials using other indices (13.8%) and 241,434 without any indices as other terms (84.8%).

### Interpretability and Active Trials

For the 80,290 active trials, 56, 145, and 696 included BMI without frailty indices, frailty indices, and other indices as conditions or diseases (0.1, 0.2, and 0.9% of all active trials, respectively). There were 79,393 active trials not involving any indices or composite measures (98.9%). There were 1,247, 144, and 13,772 active trials adopting BMI without frailty indices, frailty indices, and other indices as other terms (1.6, 0.2, and 17.2% of all active trials, respectively). There were 65,127 active trials not including any indices (81.1%).

### Interpretability and Completed Trials

Among 151,605 completed trials, 153, 105, and 1,317 included BMI without frailty indices, frailty indices, and other indices as conditions or diseases (0.1, 0.07, and 0.8% of all completed trials, respectively). There were 150,030 completed trials did not involve any indices or composite measures as conditions or diseases (98.96%). There were 2,154, 189, and 18,853 completed trials that included BMI without the use of frailty indices, frailty indices, and any other indices as other terms (1.4, 0.1, 12.4% of all completed trials, respectively). There were 130,409 completed or terminated trials not involving any indices or composite measures (86.0% of all completed or terminated trials).

### Interpretability and Terminated Trials

Among 16,052 terminated trials, 7, 6, and 88 included BMI without frailty indices, frailty indices, and other indices as conditions or diseases (0.04, 0.04, and 0.6% of all completed or terminated trials, respectively). There were 15,951 terminated trials that did not involve any indices or composite measures as conditions or diseases (99.4%). There were 116, 13, and 2,334 completed trials that included BMI without the use of frailty indices, frailty indices, and any other indices as other terms (0.7, 0.1, and 14.5% of all terminated trials, respectively). There were 13,589 terminated trials not involving any indices or composite measures (84.7% of all completed or terminated trials).

### Interpretability and Trials of Other Statuses

For the 36,153 trials of other statuses, 38, 35, and 354 included BMI without frailty indices, frailty indices, and other indices as conditions or diseases (0.1, 0.1, and 1.0% of all trials of other statuses, respectively). There were 35,726 trials of other statuses not involving any indices or composite measures (98.8%). There were 389, 22, and 4,235 active trials adopting BMI without frailty indices, frailty indices, and other indices as other terms (1.1, 0.1, and 11.7% of all trials of other statuses, respectively). There were 31,507 trials of unknown status not including any indices (87.2%).

### Use of Indices in Different Types of Trials

There was an increasing number of clinical trials, with 80,290 active trials vs. 167,657 completed or terminated trials since the initiation of the clinical trial registry. The distribution of three types of indices used was not the same across terminated, completed trials, and trials of other statuses (Chi-squared = 35.6 and 164.5, respectively, *p* < 0.001 for conditions/diseases and other terms).

### Multinomial Logit Regression

In Table 2, the ORs of using indices of different interpretability for diseases/conditions or other terms were listed. Compared to completed trials, using indices of fair-to-poor and unclear interpretability in clinical trials was associated with early termination (OR = 3.4 and 2.7, 95% CI = 1.3–8.8 and 1.3–5.8, respectively). The use of indices of other interpretability or no use of any indices was not associated with trial termination (*p* > 0.05 for all).

**Table 2:** The odds ratios of index interpretability by trial statuses.

	**Interpretability**	**Terminated trials**			**Trials of other statuses**		
		**(ORs) (baseline)**	**(95% CIs)**	***p***	**(ORs) (baseline)**	**(95% CIs)**	***p***
Diseases or conditions	Good	1.25	(0.41–3.82)	0.70	1.33	(0.78–2.29)	0.30
	Fair to poor	1.46	(0.67–3.21)	0.34	1.16	(0.79–1.71)	0.45
	Unclear	1.27	(0.58–2.77)	0.55	0.83	(0.56–1.21)	0.33
Other terms	Good	3.38	(1.30–8.80)	0.01	1.15	(0.63–2.08)	0.66
	Fair to poor	2.71	(1.27–5.78)	0.01	0.95	(0.66–1.38)	0.80
	Unclear	2.29	(1.08–4.89)	0.03	0.37	(0.25–0.53)	<0.0001
Not applicable

*Not applicable = no index or composite measure used in the trials, OR = odds ratio. Other statuses including “suspended,” “withdrawn,” and “unknown.”*

Compared to completed trials, not using any indices or composite measures in clinical trials was associated with a decreased likelihood of trial suspension, withdrawal, or unknown status (OR = 0.37, 95% CI = 0.25–0.53). The use of indices of any interpretability was not associated with trial suspension, withdrawal, or unclear status (*p* > 0.05 for all).

## Discussion

There are several intriguing findings worthy of further investigation. First, not using any indices or composite measures is associated with a decreased likelihood of trial suspension, withdrawal, or unknown status. The reasons why clinical trials may fail have been discussed from many aspects, including measurement error, statistical power, and lack of efficacy (28–30). To our knowledge, our finding is the first to support the hypothesis that the use of indices and composite measures of different interpretability in clinical trials may be associated with the rates of trial completion or early termination. There are several reasons that link indices and trial failure. Indices are possible sources of measurement errors and an important factor for inferior outcome predictability (1, 7). Indices themselves can also serve as illusory outcomes (1, 16). The correlation between index use and trial failure needs to be further investigated. We will assess this causation problem in the future.

Second, the recent and active trials are increasingly using frailty indices whether for conditions/diseases or other terms. This shows the researchers' interest in novel opportunities to improve aging and human health (1). However, three commonly used frailty indices have been found to represent the frailty theories very poorly (1). There are also conflicts between frailty theories regarding assumptions and variable selection (1). Although, some researchers have been criticizing the use of vaguely defined frailty indices (31), those that are likely to have fair-to-poor interpretability remain frequently used. This may place patients at risk for receiving unnecessary or even harmful interventions (1). To prevent harms to patients, we suggest further examination of the interpretability of the indices in trials based on published guidelines (1, 7).

Lastly, our research method using *ClinicalTrials.gov* provides a simple and feasible framework for future application. This study uses a classic epidemiologic method (32) to tabulate the distribution of clinical trials based on the use of indices of different interpretability. With the advances in text mining and machine learning (33, 34), we will continue using this method to screen other significant factors to improve the design of clinical trials.

### Limitations

Although, this study uses one of the most important sources of clinical trial information, *ClinicalTrials.gov*, limitations still remain. First, it is unclear how these indices are used in the trials, especially for the trials that include indices or composite measures in other terms. Second, the trials of other status are those suspended, withdrawn, and of unknown status. Other completed or terminated trials may not be successful in proving significant association between interventions and outcomes. Other advanced text mining methods may be required to further refine the definition of trial failure. Third, there are other important clinical trials registries to be studied (35). We think this study demonstrates the feasibility of analyzing trial databases. Fourth, indices are not used in a majority of the trials. There are relatively few numbers of trials adopting indices. We will continue this analysis with more trials in the future. Fifth, frailty indices might not be an ideal measure of interpretability. We plan to test other terms for measuring interpretability. Lastly, one related issue is that clinical trial registration is not fully complied (36). There are still trials that can't be searched in public repositories.

## Conclusion

Indices or composite measures of different interpretability have been used in clinical trials. Not using any indices or composite measures is associated with a decreased likelihood of trial suspension, withdrawal, or unknown statuses. The use of indices of fair-to-poor or unclear interpretability is associated with early trial termination. The proportions of using frailty indices are increasing in active trials as conditions/diseases or other terms, compared to completed or terminated trials. Based on the findings, we hypothesize that using indices of poor interpretability may lead to trial failure. We will further test the hypothesis that the use of indices of inadequate interpretability causes trial failure and continue applying this research method to improve clinical trial design.

## Data Availability Statement

The datasets generated for this study are available on request to the corresponding author.

## Author Contributions

Y-SC conceptualized the research project, restructured the data, conducted the statistical analyses, and drafted the manuscripts. C-JW, H-CW, W-CC, and DM reviewed the manuscript and provided constructive comments. All authors contributed to the article and approved the submitted version.

## Conflict of Interest

Y-SC and DM are currently employed by the Canadian Agency for Drugs and Technologies in Health. The remaining authors declare that the research was conducted in the absence of any commercial or financial relationships that could be construed as a potential conflict of interest.

## Publisher's Note

All claims expressed in this article are solely those of the authors and do not necessarily represent those of their affiliated organizations, or those of the publisher, the editors and the reviewers. Any product that may be evaluated in this article, or claim that may be made by its manufacturer, is not guaranteed or endorsed by the publisher.

## References

[1] Chao Y-S Wu H-C Wu C-J Chen W-C. Index or illusion: the case of frailty indices in the health and retirement study. PLoS ONE. (2018) 13:e0197859. 10.1371/journal.pone.0197859 30020923 PMC6051600

[2] Rockwood K Andrew M Mitnitski A. A comparison of two approaches to measuring frailty in elderly people. J Gerontol A Biol Sci Med Sci. (2007) 62:738–43. 10.1093/gerona/62.7.738 17634321

[3] Rockwood K Song X MacKnight C Bergman H Hogan DB McDowell I. A global clinical measure of fitness and frailty in elderly people. CMAJ. (2005) 173:489–95. 10.1503/cmaj.050051 16129869 PMC1188185

[4] Rockwood K Abeysundera MJ Mitnitski A. How should we grade frailty in nursing home patients? J Am Med Dir Assoc. (2007) 8:595–603. 10.1016/j.jamda.2007.07.012 17998116

[5] Searle SD Mitnitski A Gahbauer EA Gill TM Rockwood K. A standard procedure for creating a frailty index. BMC Geriatrics. (2008) 8:24. 10.1186/1471-2318-8-24 18826625 PMC2573877

[6] Chao Y-S McGolrick D Wu C-J Wu H-C Chen W-C. A proposal for a self-rated frailty index and status for patient-oriented research. BMC Res Notes. (2019) 12:172. 10.1186/s13104-019-4206-3 30909969 PMC6434809

[7] Chao Y-S Wu C-J. Principal component-based weighted indices and a framework to evaluate indices: results from the medical expenditure panel survey 1996 to 2011. PLoS ONE. (2017) 12:e0183997. 10.1371/journal.pone.0183997 28886057 PMC5590867

[8] Cigolle CT Ofstedal MB Tian Z Blaum CS. Comparing models of frailty: the health and retirement study. J Am Geriatr Soc. (2009) 57:830–9. 10.1111/j.1532-5415.2009.02225.x 19453306

[9] Fried LP Tangen CM Walston J Newman AB Hirsch C Gottdiener J. Frailty in older adults: evidence for a phenotype. J Gerontol A Biol Sci Med Sci. (2001) 56:M146–57. 10.1093/gerona/56.3.M146 11253156

[10] Rockwood K Mitnitski A. Frailty in relation to the accumulation of deficits. J Gerontol Biol Sci Med Sci. (2007) 62:722–7. 10.1093/gerona/62.7.722 17634318

[11] Fried LP Ferrucci L Darer J Williamson JD Anderson G. Untangling the concepts of disability, frailty, and comorbidity: implications for improved targeting and care. J Gerontol A Biol Sci Med Sci. (2004) 59:M255–63. 10.1093/gerona/59.3.M255 15031310

[12] Mitnitski A Xiaowei S Skoog I Broe GA Cox JL Grunfeld E. Relative fitness and frailty of elderly men and women in developed countries and their relationship with mortality. J Am Geriatr Soc. (2005) 53:2184–9. 10.1111/j.1532-5415.2005.00506.x 16398907

[13] Fedarko NS. The biology of aging and frailty. Clin Geriatr Med. (2011) 27:27–37. 10.1016/j.cger.2010.08.006 21093720 PMC3052959

[14] Chao Y-S Lin K-F Wu C-J Wu H-C Hsu H-T Tsao L-C. Simulation study to demonstrate biases created by diagnostic criteria of mental illnesses: major depressive episodes, dysthymia, and manic episodes. BMJ Open. (2020) 10:e037022. 10.1136/bmjopen-2020-037022 33172939 PMC7656951

[15] Barnwell-Menard JL Li Q Cohen AA. Effects of categorization method, regression type, and variable distribution on the inflation of Type-I error rate when categorizing a confounding variable. Stat Med. (2015) 34:936–49. 10.1002/sim.6387 25504513

[16] Chao Y-S Wu C-J Wu H-C Hsu H-T Tsao L-C Cheng Y-P. Composite diagnostic criteria are problematic for linking potentially distinct populations: the case of frailty. Sci Rep. (2020) 10:2601. 10.1038/s41598-020-58782-1 32054866 PMC7018968

[17] Chao Y-S Wu H-C Wu C-J Chen W-C. Stages of biological development across age: an analysis of canadian health measure survey 2007–2011. Front Public Health. (2018) 5:355. 10.3389/fpubh.2017.00355 29376046 PMC5768641

[18] Kahn R. Metabolic syndrome-what is the clinical usefulness? Lancet. (2008) 371:1892–3. 10.1016/S0140-6736(08)60731-X 18501420

[19] Aguilar-Salinas CA Rojas R Gomez-Perez FJ Franco A Olaiz G Rull JA. [The metabolic syndrome: a concept in evolution]. Gac Med Mex. (2004) 140(Suppl. 2):S41–8. 15641471

[20] Ford ES Giles WH Dietz WH. Prevalence of the metabolic syndrome among us adults: findings from the third national health and nutrition examination survey. JAMA. (2002) 287:356–9. 10.1001/jama.287.3.356 11790215

[21] Sattar N McConnachie A Shaper AG Blauw GJ Buckley BM de Craen AJ. Can metabolic syndrome usefully predict cardiovascular disease and diabetes? Outcome data from two prospective studies. Lancet. (2008) 371:1927–35. 10.1016/S0140-6736(08)60602-9 18501419

[22] U.S. National Library of Medicine. ClinicalTrials.gov Background. Washington, DC: U.S. National Library of Medicine (2018). Available online at: https://clinicaltrials.gov/ct2/about-site/background (accessed Febraury 14, 2018).

[23] U.S. National Library of Medicine. Glossary of Common Site Terms. Washington, DC: U.S. National Library of Medicine (2017). Available online at: https://clinicaltrials.gov/ct2/about-studies/glossary (accessed Febraury 1, 2018).

[24] Chao Y-S Wu C-J. PP46 when composite measures or indices fail: data processing lessons. Int J Technol Assess Health Care. (2019) 34(Suppl. 1):83. 10.1017/S0266462318002088

[25] Jurka TP. Maxent: an R package for low-memory multinomial logistic regression with support for semi-automated text classification. R J. (2012) 4:56–9. 10.32614/RJ-2012-007

[26] R Development Core Team. R: A Language and Environment for Statistical Computing. Vienna: R Foundation for Statistical Computing (2016).

[27] RStudio Team. RStudio: Integrated Development for R. Boston, MA: RStudio, Inc. (2016).

[28] Perkins DO Wyatt RJ Bartko JJ. Penny-wise and pound-foolish: the impact of measurement error on sample size requirements in clinical trials. Biol Psychiatry. (2000) 47:762–6. 10.1016/S0006-3223(00)00837-4 10773186

[29] Kobak KA Kane JM Thase ME Nierenberg AA. Why do clinical trials fail?: The problem of measurement error in clinical trials: time to test new paradigms? J Clin Psychopharmacol. (2007) 27:1–5. 10.1097/JCP.0b013e31802eb4b7 17224705

[30] Bone RC. Why sepsis trials fail. JAMA. (1996) 276:565–6. 10.1001/jama.1996.03540070061032 8709407

[31] Bergman H Ferrucci L Guralnik J Hogan DB Hummel S Karunananthan S. Frailty: an emerging research and clinical paradigm–issues and controversies. J Gerontol A Biol Sci Med Sci. (2007) 62:731–7. 10.1093/gerona/62.7.731 17634320 PMC2645660

[32] Rothman KJ Greenland S Lash TL. Modern Epidemiology. New York, NY: Wolters Kluwer Health/Lippincott Williams & Wilkins (2008).

[33] James G Witten D Hastie T Tibshirani R. An Introduction to Statistical Learning: with Applications in R. New York, NY: Springer (2013). 10.1007/978-1-4614-7138-7

[34] Hastie T Tibshirani R Friedman J. The Elements of Statistical Learning: Data Mining, Inference, and Prediction. 2nd ed. New York, NY: Springer (2009).

[35] Dickersin K Rennie D. Registering clinical trials. JAMA. (2003) 290:516–23. 10.1001/jama.290.4.516 12876095

[36] Huser V Cimino JJ. Evaluating adherence to the International Committee of Medical Journal Editors' policy of mandatory, timely clinical trial registration. J Am Med Inform Assoc. (2013) 20:e169–74. 10.1136/amiajnl-2012-001501 23396544 PMC3715364

